# Protein kinase C regulates AMPA receptor auxiliary protein Shisa9/CKAMP44 through interactions with neuronal scaffold PICK1

**DOI:** 10.1002/2211-5463.12261

**Published:** 2017-08-15

**Authors:** Stella‐Amrei Kunde, Nils Rademacher, Hanna Zieger, Sarah A. Shoichet

**Affiliations:** ^1^ Neuroscience Research Center/Institute of Biochemistry Charité – Universitätsmedizin Berlin Germany

**Keywords:** AMPAR auxiliary protein, molecular neurobiology, phosphorylation, PKC, postsynaptic proteins

## Abstract

Synaptic α‐amino‐3‐hydroxyl‐5‐methyl‐4‐isoxazole‐propionate (AMPA) receptors are essential mediators of neurotransmission in the central nervous system. Shisa9/cysteine‐knot AMPAR modulating protein 44 (CKAMP44) is a transmembrane protein recently found to be present in AMPA receptor‐associated protein complexes. Here, we show that the cytosolic tail of Shisa9/CKAMP44 interacts with multiple scaffold proteins that are important for regulating synaptic plasticity in central neurons. We focussed on the interaction with the scaffold protein PICK1, which facilitates the formation of a tripartite complex with the protein kinase C (PKC) and thereby regulates phosphorylation of Shisa9/CKAMP44 C‐terminal residues. This work has implications for our understanding of how PICK1 modulates AMPAR‐mediated transmission and plasticity and also highlights a novel function of PKC.

**Abbreviations**
AMPA: α‐amino‐3‐hydroxyl‐5‐methyl‐4‐isoxazole‐propionate
CaMKII: Ca^2+^/calmodulin‐dependent protein kinase II
CKAMP44: cysteine‐knot AMPAR modulating protein 44
MAGI1: membrane‐associated guanylate kinase inverted 1
MAGUK: membrane‐associated guanylate kinase
nPIST: neuronal protein interacting specifically with Tc10
PDZ: PSD‐95 Dlg1 and ZO‐1
PICK1: protein interacting with C kinase 1
PKA: protein kinase A
PKC: protein kinase C
PMA: phorbol‐12‐myristate‐13‐acetate
PSD‐95: postsynaptic density protein 95
SAP102: synapse‐associated protein 102
TARP: transmembrane AMPAR regulatory protein
Y2H: yeast two‐hybrid

The ionotropic glutamate receptors are of particular importance for fast synaptic transmission in the central nervous system. Among these ligand‐gated receptors, the α‐amino‐3‐hydroxyl‐5‐methyl‐4‐isoxazole‐propionate (AMPA) receptors are especially important for regulating changes in synaptic strength; elucidating the mechanisms by which these receptors are regulated is therefore critical for our molecular understanding of information storage in the brain. Recent studies indicate that they are found in complex with additional transmembrane proteins that modulate receptor function, including the Shisa/cysteine‐knot AMPAR modulating protein (CKAMP) family of proteins, among others.

Shisa/CKAMP proteins were originally described for their role in head formation 1 and were named *Shisa* after a form of sculpture with a large head (similar to the Egyptian Sphinx) that is found in southern Japan. The first study on *Shisa* family transcript expression in the mouse brain focussed on the transcript for *Shisa2*; since then, high‐throughput *in situ* analyses have confirmed that several of the *Shisa* transcripts, including. *Shisa5, 6, 7* and *9,* exhibit high expression in the brain, and each one has restricted expression in a subset of CNS neurons (see Allen Mouse Brain Atlas: http://mouse.brain-map.org) 2, suggesting that these proteins may be involved in specific neuronal functions. Indeed, more recent studies indicated that Shisa9 is present in AMPA receptor complexes and that its presence there regulates synaptic transmission by modulating AMPA receptor channel properties 3. In addition, it has now been shown that not only Shisa9 but also Shisa6, 7 and 8 are capable of influencing receptor function 4, 5. In the light of these new studies, this subgroup of Shisa proteins is now also referred to as the CKAMP family, based on their common ability to modulate AMPAR synaptic transmission. Another property that these CKAMP proteins share is the presence of a conserved C‐terminal sequence typical for proteins that have been shown to bind a postsynaptic density protein 95 (PSD‐95) Dlg1 and ZO‐1 (PDZ) domain, the 80–90 AA structurally conserved motif originally identified for its presence in PSD‐95, Dlg1 and ZO‐1 proteins.

Numerous cytosolic proteins at synapses harbour PDZ domains and are capable of interacting directly with the cytoplasmic tails of transmembrane receptors, and it has been shown that such PDZ–ligand interactions are important for the trafficking of AMPA receptors to and from the cell surface 6. Notably, it has become clear in recent years that direct interactions between PDZ domain proteins and AMPA receptor subunits themselves are not exclusively responsible for this regulation of AMPAR surface expression. Cumulative data from several groups suggest that transmembrane AMPAR‐interacting proteins are essential for this function (for review, see 7). As several of these proteins also influence the gating properties of isolated AMPA receptors, they are often referred to collectively as AMPA receptor auxiliary proteins (for recent reviews, see 7, 8). Among these transmembrane AMPAR auxiliary proteins, the transmembrane AMPAR regulatory protein (TARP) family has been studied in most depth: it is well established that TARPs are involved in the modulation of AMPAR desensitisation and also play a critical role in AMPAR trafficking to the surface. Importantly, the latter function of TARPs clearly relies on the C‐terminal cytosolic region that binds to PDZ domains of specific synaptic scaffold molecules 9, 10, 11, illustrating that the cytosolic C‐terminal tail of AMPAR auxiliary proteins can play a decisive role in AMPA receptor function via this mechanism.

It has further been established that phosphorylation of the C‐terminal regions of ionotropic glutamate receptor subunits themselves, and also the TARP family of auxiliary proteins, can dramatically influence receptor function, presumably by influencing interactions between AMPAR complexes and cytosolic proteins or specific cellular components. It has been shown, for example, that serine residues within the C‐terminal cytosolic region of AMPAR subunits GluA1 and GluA2 are phosphorylated [by, e.g., protein kinase A (PKA), PKC and Ca^2+^/calmodulin‐dependent protein kinase II (CaMKII)] and that the phosphorylation status influences channel conductance and opening probability (for recent review, see 12). Also relevant is the fact that modulating the phosphorylation status of the TARPɣ2 C terminus can dramatically influence its localisation and function 13, 14, 15.

In this study, we focus on Shisa9/cysteine‐knot AMPAR modulating protein 44 (CKAMP44), a small protein with only one transmembrane domain (TM). It was initially proposed that its effects on AMPAR‐mediated transmission are mediated by a set of six N‐terminal extracellular cysteines, as this region resembles that of the snail conotoxin (Cys‐ikot‐ikot) that disrupts AMPAR desensitisation 3. It has since been shown that the C‐terminal intracellular domain is also functionally important. Like the TARPs, Shisa9/CKAMP44 harbours a long C‐terminal cytosolic tail that is presumably a target for both interactions with cytosolic scaffolds and regulation by associated kinases. Given that Shisa9/CKAMP44 binds to AMPARs and modulates specific functions at the membrane, we considered it highly relevant to explore how this protein is regulated by other protein–protein interactions. In this study, we have screened for and identified cytoplasmic interaction partners of Shisa9/CKAMP44, including the synaptic scaffold molecule protein interacting with C kinase 1 (PICK1). More importantly, we demonstrate that this interaction facilitates regulation of Shisa9/CKAMP44 by PKC, which highlights a novel function of PKC in neurons that will significantly influence future investigations into PICK1 and PKC‐mediated effects on AMPAR composition and function.

## Results

### Shisa9/CKAMP44 interacts with several synapse‐associated PDZ‐containing scaffold proteins

**Figure 1:**
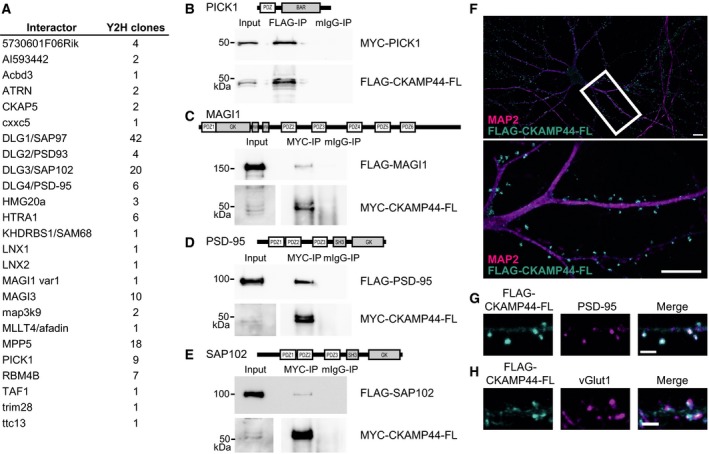
Novel interaction partners of Shisa9/CKAMP44 and localisation at synaptic sites. (A) Results of a Y2H screen with Shisa9/CKAMP44 (mouse, AAs 171–424, cytoplasmic tail), as an N‐LexA‐Shisa9‐C fusion, performed on a mouse brain cDNA library. (B–E) Confirmation of selected interactions, namely those with PICK1, MAGI1, PSD‐95 and SAP102: coimmunoprecipitation experiments (COS‐7) of overexpressed FLAG‐ or MYC‐tagged full‐length Shisa9/CKAMP44 (FLAG/MYC‐CKAMP44‐FL) together with MYC‐PICK1 (B), FLAG‐MAGI1 (C), FLAG‐PSD‐95 (D) or FLAG‐SAP102 (E). Proteins were immunoprecipitated with the antibodies indicated, that is with commercially available FLAG or MYC antibodies, or with mIgG as a negative control, and subsequently detected by WB. Input (lysate) control is shown on the left (longer exposures in C/D/E). (F) Overlay image of typical primary hippocampal neuron (DIV21) expressing FLAG‐CKAMP44‐FL (cyan); neurons are costained for MAP2 (magenta); high‐resolution insert shown below. Scale bar 10 μm. (G) FLAG‐CKAMP44‐FL (cyan, left) colocalises with endogenous PSD‐95 (magenta, middle), a postsynaptic protein. Overlay is shown on the right. Scale bar 2 μm. (H) Costaining of FLAG‐CKAMP44‐FL (cyan, left) with endogenous presynaptic vGlut1 (magenta, middle) shows adjacent signals. Overlay is shown on the right. Scale bar 2 μm.

In order to determine putative cytosolic binding partners of Shisa9/CKAMP44, we used this region of the protein as bait in a yeast two‐hybrid (Y2H) assay and identified several putative interaction partners (summarised in Fig. 1A). Within this list were several scaffold proteins harbouring PDZ domains [e.g. PICK1, DLG1/SAP97, DLG2/PSD‐93, DLG3/synapse‐associated protein 102 (SAP102), DLG4/PSD‐95, MPP5, membrane‐associated guanylate kinase inverted 1 (MAGI1), MAGI3, LNX1, LNX2 and HTRA1], suggesting that the C‐terminal region of Shisa9/CKAMP44 is indeed a PDZ ligand, as predicted by sequence analysis 3 and likewise observed in more recent work 5, 16, 17. We validated an interaction of the synapse‐associated PDZ domain proteins PICK1, MAGI1, PSD‐95 and SAP102 with the full‐length Shisa9/CKAMP44 by coimmunoprecipitation (Fig. 1B–E), and confirmed that Shisa9/CKAMP44 localises to synaptic sites in primary neurons (Fig. 1F–H). Further experiments indicated that the interaction with membrane‐associated guanylate kinase (MAGUK) scaffolds relies exclusively on the C‐terminal PDZ‐binding motif of Shisa9/CKAMP44 and the two most N‐terminal MAGUK PDZ domains (see Fig. S1), highlighting a clear binding specificity for PDZ domains 1 and 2.

### The Shisa9/CKAMP44 interaction with PICK1 facilitates Shisa9/CKAMP44 phosphorylation by PKC

**Figure 2:**
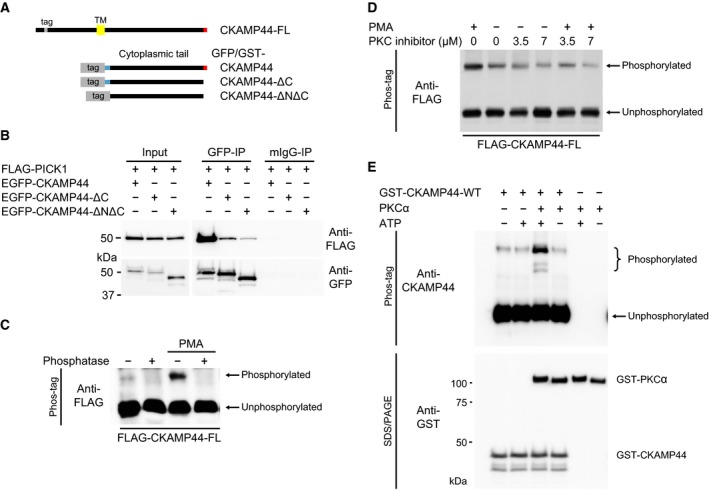
Shisa9/CKAMP44 interacts with PICK1 and is phosphorylated by PKCα. (A) Schematic overview of Shisa9/CKAMP44 constructs used in this study (including the location of the respective tag): in CKAMP44‐FL (full length), the tag is C‐terminal to the signal peptide in the extracellular domain (TM, yellow). Constructs with only the cytoplasmic tail of Shisa9/CKAMP44 are N‐terminally tagged (with either EGFP or GST). PDZ ligand sequences are shown in red and are deleted in constructs labelled with ΔC (PDZ‐binding‐deficient variant). A conserved region of Shisa9/CKAMP44 (blue; N‐terminal region of the cytoplasmic tail) is deleted in the construct CKAMP44‐ΔNΔC. Constructs are drawn to scale. (B) Comparative coimmunoprecipitation experiments of PICK1 (FLAG‐PICK1) with Shisa9/CKAMP44 C‐terminal variants EGFP‐CKAMP44, −ΔC, or −ΔNΔC). Proteins were precipitated using GFP antibody or mIgG (negative control) and detected using the antibodies indicated. Inputs are shown on the left. (C) Full‐length Shisa9/CKAMP44 (FLAG‐CKAMP44‐FL), expressed in COS‐7 cells, is phosphorylated following induction by PMA (1 μm, 30 min). Phosphorylated proteins (indicated) are separated from nonphosphorylated proteins (indicated) via Phos‐tag‐SDS/PAGE and observed via WB; phosphatase treatment with alkaline phosphatase is used as a negative control, thereby highlighting the phosphorylation dependence of the mobility shift. (D) Phosphorylation of Shisa9/CKAMP44 (FLAG‐CKAMP44‐FL) is induced with PMA and inhibited by application of the PKC inhibitor GF109203X in COS‐7 cells. Again phosphorylated proteins are observable via Phos‐tag‐SDS/PAGE. PKC inhibitor concentrations are indicated above. (E) An *in vitro* PKCα kinase assay followed by Phos‐tag SDS/PAGE analysis highlights a PKCα‐dependent mobility shift of the phosphorylated substrate GST‐CKAMP44 (top panel, Phos‐tag SDS/PAGE). Presence of PKCα and kinase substrate present in the same samples is shown below (lower panel, standard Laemmli SDS/PAGE and WB; antibodies indicated).

In the following experiments, we focussed on the interaction of Shisa9/CKAMP44 with the protein PICK1, which has been shown previously to bind directly with AMPA receptor subunit C‐terminal tails in a ligand–PDZ domain interaction 18, 19. Unexpectedly, we observed that the Shisa9/CKAMP44‐PICK1 interaction is not exclusively dependent on the PDZ‐binding motif in Shisa9/CKAMP44, which differs from the interaction with PSD‐95 and SAP102 (Fig. S1) as demonstrated in coimmunoprecipitation experiments (Fig. 2B). To further narrow down the interaction region, we deleted a more proximal region of the cytosolic tail and observed an even further reduced interaction of PICK1 with Shisa9/CKAMP44 (Fig. 2B; for schematic diagram of deletion constructs used, see Fig. 2A). This proximal region of the cytoplasmic tail of Shisa9/CKAMP44 contains an amino acid (AA) stretch that is highly conserved between species and also across Shisa/CKAMP family proteins 4 and has been shown to bind GluA1 in co‐immunoprecipitation (IP) experiments 17. In this context, it is interesting that this region also has an influence on PICK1‐Shisa9/CKAMP44 binding.

**Figure 3:**
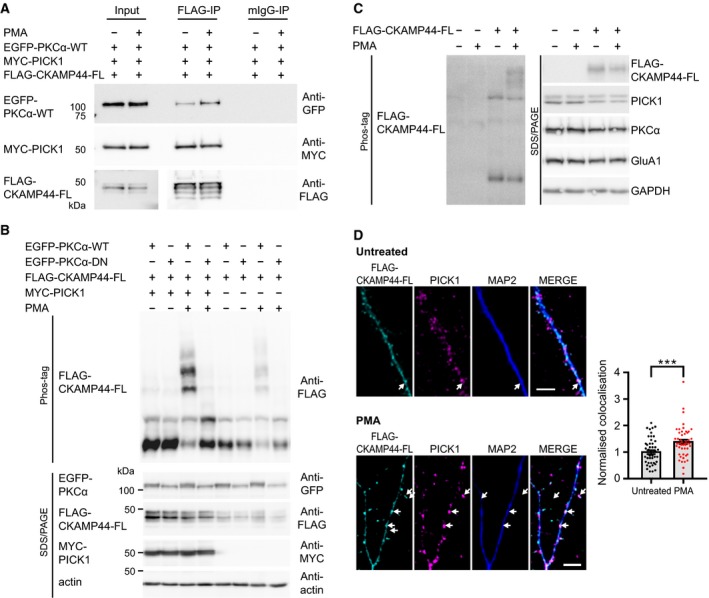
Shisa9/CKAMP44‐PICK1 complex formation and regulation by active PKC in neurons. (A) PKC activation status influences CKAMP44/PICK1/PKCα complex formation: coimmunoprecipitation experiments following expression of Shisa9/CKAMP44 (FLAG‐CKAMP44‐FL), PICK1 (MYC‐PICK1) and PKCα (EGFP‐PKCα‐WT) in COS‐7 cells indicate that the three proteins form a complex. After pull‐down FLAG‐CKAMP44‐FL, coprecipitated proteins are detected by WB, as indicated. mIgG serve as a negative pull‐down control. Comparison (with or without PMA activation, 1 μm, 30 min) indicates that EGFP‐PKCα association with FLAG‐CKAMP44‐FL/MYC‐PICK1 protein complex is modulated by PKC activation status. (B) Phosphorylation of overexpressed Shisa9/CKAMP44 (FLAG‐CKAMP44‐FL) in COS‐7 cells in the presence of either wild‐type or kinase‐dead PKCα (EGFP‐PKCα‐WT and EGFP‐PKCα‐DN; compare lanes 3 and 4) with or without PICK1 (MYC‐PICK1; compare lanes 3 and 7) is shown (top panel; Phos‐tag: phosphorylated proteins observed by mobility shift). PKC was activated by PMA (1 μm, 30 min); controls for relevant protein levels (actin: loading control) are shown below (Laemmli SDS/PAGE and WB). Antibodies used are indicated on the right. (C) In primary hippocampal neurons, activation of PKC by PMA (0.2 μm, 15 min) likewise affects Shisa9/CKAMP44 phosphorylation status (compare lanes 3 and 4 in the Phos‐tag blot, left panel). Shisa9/CKAMP44 proteins were observed by WB following viral transduction of FLAG‐CKAMP44‐FL, with or without activation by PMA. Neurons were harvested at DIV22; on the right, control levels of related proteins are observed via SDS/PAGE. (D) Overlapping localisation of Shisa9/CKAMP44 and PICK1 in primary hippocampal neurons after activation of PKC: neurons were infected (DIV 10–15) with virus expressing Shisa9/CKAMP44 (FLAG‐CKAMP44‐FL) and fixed at DIV 20–24 without a treatment (upper panel) or after activation of PKC by PMA (0.2 μm, 15 min; lower panel). Immunofluorescence with a FLAG antibody (cyan), an antibody to endogenous PICK1 (magenta) and an antibody to endogenous MAP2 (blue) is shown. Shisa9/CKAMP44–PICK1 overlapping puncta are indicated. Merged images are shown on the right. Scale bar 5 μm. The colocalisation of FLAG‐CKAMP44‐FL and endogenous PICK1 in rat hippocampal neurons was quantified with the Puncta Analyzer (imagej plugin) in dendritic segments of FLAG‐CKAMP44‐FL‐infected neurons (untreated: *n* = 49 neurons from *N* = 4 cultures; PMA treated: *n* = 48 neurons from *N* = 4 cultures) and normalised to the mean of untreated condition. The colocalisation increased significantly after PMA treatment (0.2 μm PMA for 15 min) by the factor of 1.388 ± 0.086 (*** *P* = 0.0008, two‐tailed Mann–Whitney *U*‐test, *U* = 716.5) compared to untreated neurons (1.0 ± 0.074). Data are represented as mean ± SEM.

These data led us to hypothesise that the binding between PICK1 and Shisa9/CKAMP44 is an important, high‐affinity interaction that could be subject to temporal or signal‐dependent regulation. PICK1 was named for its role as an interaction partner for the protein kinase PKC 20, 21. We therefore asked whether Shisa9/CKAMP44 might be a target of PKC‐mediated phosphorylation. Using the Phos‐tag system, we demonstrated that wild‐type Shisa9/CKAMP44 is indeed modified following expression in heterologous cells and that the observed mobility shift was inhibited following phosphatase treatment (Fig. 2C), confirming that we indeed observed a phosphorylation‐dependent shift. In addition, phosphorylation could be modulated using phorbol‐12‐myristate‐13‐acetate (PMA), which activates PKC signal cascades. Moreover, while PMA treatment amplified the relative phospho‐Shisa9/CKAMP44 signals, PKC inhibitors, including GF109203X, reduced total phosphorylation of Shisa9/CKAMP44 (Fig. 2C,D) in this system, supporting the idea that Shisa9/CKAMP44 can be regulated by PKC. Importantly, we also observe these PKC‐dependent changes in the phosphorylation of Shisa9/CKAMP44 in a neuronal environment (see Fig. 3), and we demonstrated that Shisa9/CKAMP44 is indeed a direct PKC target using purified PKCα with the GST‐tagged Shisa9/CKAMP44 cytosolic tail as a substrate in an *in vitro* kinase assay (Fig. 2E).

### Shisa9/CKAMP44 forms a complex with PICK1 and PKC that is regulated by activation of PKC signalling cascades in neurons

We next confirmed that Shisa9/CKAMP44, PICK1 and PKC indeed form a tripartite complex in coimmunoprecipitation experiments (Fig. S2). Interestingly, activated PKC binds more effectively than inactive PKC to the PICK1‐Shisa9/CKAMP44 complex (Fig. 3A; co‐IP with or without PMA stimulation), which is in line with the dramatic phosphorylation of Shisa9/CKAMP44 observed following PMA stimulation in the presence of both PICK1 and wild‐type PKCα (Fig. 3B; lane 3 of Phos‐tag panel). A similar phospho‐dependent mobility shift of Shisa9/CKAMP44 in response to PKC activation was observed in primary hippocampal neurons (Fig. 3C; compare lanes 3 and 4 of the Phos‐tag panel), confirming the importance of endogenous PKC in the regulation of this AMPAR auxiliary protein at synapses. In line with these data, we observe a strong increase in colocalisation of neuronal PICK1 with synaptic Shisa9/CKAMP44 following activation of endogenous PKC with PMA (see representative images in Fig. 3D). Digital analysis of overlapping signals (synaptic FLAG‐CKAMP44‐FL with endogenous PICK1 puncta) indicated approximately a 1.4‐fold increase in colocalisation in response to treatment with PMA (1.388 ± 0.086; see data points and quantification of observations in the Fig. 3D bar diagram).

**Figure 4:**
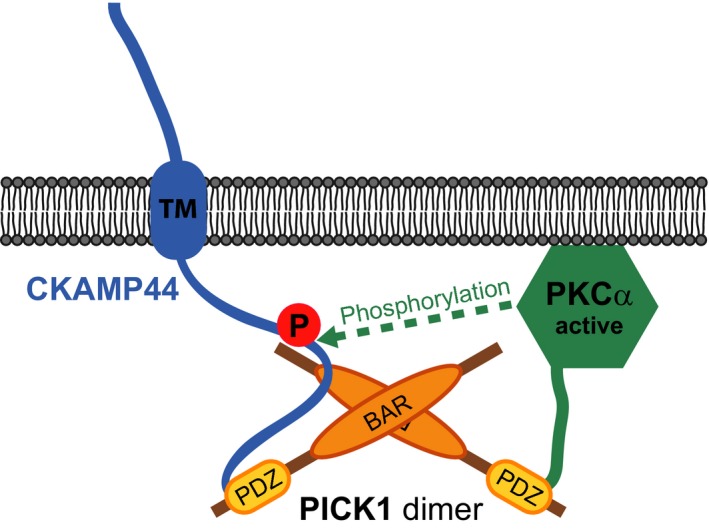
A model illustrating the Shisa9/CKAMP44–PICK1‐PKCα protein complex. PICK1 can dimerise and is likely able to bind Shisa9/CKAMP44 and PKCα at the same time, thereby enabling PKCα‐mediated phosphorylation of Shisa9/CKAMP44 upon kinase activation.

These data support a model proposed earlier 19, in which activation of PKC stabilises the interaction between PKCα and PICK1. We further demonstrate here that PKC activation promotes localisation of PICK1 towards Shisa9/CKAMP44 at synaptic sites, which enables an inducible and dynamic PKC‐mediated regulation of the Shisa9/CKAMP44 C terminus. These novel ideas are summarised in Fig. 4.

## Discussion

In this study, we have explored the molecular interactions mediated by the cytosolic C‐terminal region of the CNS‐expressed Shisa9/CKAMP44 protein. We have identified several Shisa9/CKAMP44 binding proteins, including several scaffold molecules that play an established role in the regulation of cell–cell communication, for example PICK1 and the MAGUK family members PSD‐95, SAP102 and MAGI1. In addition to the synaptic MAGUK proteins (PDZ‐dependent interaction), the scaffold molecule PICK1 exhibited strong binding to the cytosolic region of the AMPAR auxiliary protein Shisa9/CKAMP44.

Protein interacting with C kinase 1 and protein kinase C (PKC) – as their names indicate – have an established biochemical connection, and several studies have confirmed that PICK1 cooperates with PKC to execute various regulatory functions in neurons 22, 23, 24, 25. Moreover, a role for both PICK1 and PKC in the regulation of AMPAR‐mediated plasticity has been explored 19, 26, 27, 28. In the context of these studies, our data highlighting a strong interaction between PICK1 and the AMPAR receptor auxiliary protein Shisa9/CKAMP44 and the subsequent discovery that this protein is a novel PKC target, are of interest. Our observation that the overlapping subcellular localisation of PICK1 and Shisa9/CKAMP44 in neurons is dynamically regulated by activation of PKC signalling cascades supports the idea that the interaction between these two proteins may be a target of finely tuned temporal or activity‐dependent regulation by intracellular signals. Further supporting this idea, we found that the formation of a Shisa9/CKAMP44‐PICK1‐PKC complex in response to PKC activation leads to changes in the phosphorylation status of Shisa9/CKAMP44 in primary neurons.

To date, there have been relatively few investigations into the function of Shisa9/CKAMP44, but it has been demonstrated that the N‐terminal extracellular domain of Shisa9/CKAMP44 is important for regulating AMPAR desensitisation kinetics 3, 17. In the context of our data, investigations into Shisa9/CKAMP44‐mediated modulation of AMPAR trafficking to the surface, which have so far yielded diverse results, are of particular interest. In the hippocampal CA1 region, Shisa9/CKAMP44‐deficient neurons did not exhibit a reduction in synaptic AMPARs 3; however, in dentate gyrus granule cells, where endogenous Shisa9/CKAMP44 is expressed at higher levels, studies on KO animal tissue suggest that the trafficking of AMPARs to the synaptic membrane involves both TARP and CKAMP auxiliary proteins 17. It has been shown clearly that this function of TARP family auxiliary proteins relies on the cytosolic tail 9, 10, 11; it is therefore plausible that CKAMP family members likewise participate in this process via intracellular molecular mechanisms.

Our observations that the Shisa9/CKAMP44 intracellular domain is a target of phospho‐regulation and interacts differentially with certain scaffold proteins suggest that there may be parallels between how TARPs and CKAMP family proteins influence receptor trafficking. It has been shown that phosphorylation‐dependent interactions of TARPs can regulate synaptic content of both TARPs and AMPAR subunits: the phosphorylation status of the TARPɣ2 C terminus influences its association with the membrane lipid bilayer 15 and also affects its ability to interact with PSD‐95 13, 14; these changes have implications for AMPAR‐mediated synaptic transmission. It has also been shown that – in addition to the most C‐terminal region responsible for interacting with synaptic MAGUKS – other cytosolic regions of TARPs can influence receptor trafficking 29. In addition, the interaction between TARPɣ2 and the intracellular scaffold neuronal protein interacting specifically with Tc10 (nPIST; also known as GOPC/CAL), a Golgi‐enriched protein 30, 31, can modulate the synaptic targeting of AMPAR complexes 32. In this case, it is not the C‐terminal PDZ‐binding region of TARPɣ2 that is responsible for the function but a more proximal cytosolic region close to the TM. These authors propose a mechanism that involves – early on in the secretory process – a PDZ‐independent interaction between TARPɣ2 and nPIST, which is followed by a PDZ domain‐mediated interaction with PSD‐95 that ensures synaptic localisation. Both of these interactions are important for the function of TARPs as mediators of AMPAR trafficking, and it is plausible that a similar model applies to Shisa9/CKAMP44, which is able to bind PICK1 and also PSD‐95‐family synaptic MAGUKs.

Although Shisa9/CKAMP44 clearly binds to PICK1 via its PDZ‐binding motif, we demonstrate that at least one additional region, namely the N‐terminal region of its intracellular domain, which is also important for its effects on AMPAR modulation 17, is also involved in binding to this scaffold protein. This leads to the possibility that PICK1 and GluA1 might compete for this binding region of Shisa9/CKAMP44. An additional possibility is that these interactions are temporally and/or spatially separated; for example, a brief interaction between Shisa9/CKAMP44 and PICK1 during early trafficking could be followed by PSD‐95‐guided synaptic localisation and subsequent functional interaction with AMPAR subunits.

Importantly, our observation that there are dramatic effects on the phospho‐regulation of Shisa9/CKAMP44 specifically when it is in a complex with PICK1 and PKC suggests that formation of this tripartite complex is important. The fact that the complex can be dynamically regulated suggests that it may have a transitory role, perhaps comparable to that proposed for the TARPɣ2/nPIST interaction, which is relevant at specific time points in the secretion and trafficking of receptor complexes *en route* to the membrane. Indeed, numerous investigations illustrate a role for PICK1 in both basal (e.g. see 27, 33) and activity‐dependent receptor trafficking (e.g. see 34, 35, 36, 37; for review, see 38). It is possible that the Shisa9/CKAMP44‐PICK1 interaction and subsequent PKC‐mediated Shisa9/CKAMP44 phosphorylation that we observe might influence early phases of AMPAR trafficking in a subset of excitatory neurons. Interestingly, a role for PICK1 in the export of AMPA receptors from the endoplasmic reticulum (ER) has recently been reported 39, and PKC‐mediated phosphorylation of other ionotropic glutamate receptor subunits participates in the regulation of their export from the ER 40, 41, 42. In addition, it has been shown that the interaction between PICK1 and active PKCα is transient, and primarily important for initial targeting of PICK1 to the membrane 43, where the transition of assembled AMPAR complexes to other membrane‐associated PDZ domain scaffolds could take place. Moreover, there is evidence that trans‐Golgi network‐derived organelles in the dendrites of hippocampal neurons undergo regulated exocytosis in response to calcium signals 44, further supporting a role for the calcium sensing PICK1/PKC complex in this process.

In summary, this study highlights a novel molecular mechanism that may contribute to PKC association with AMPARs: via interaction with the PICK1‐Shisa9/CKAMP44 complex. It thereby lays the groundwork for further investigations into the functional modulation of Shisa9/CKAMP44 and associated AMPAR protein complexes via PKC signalling cascades that involve PICK1.

## Materials and methods

### Y2H screen

The Y2H screen (Hybrigenics ULTImate Y2H) was performed by HYBRIGENICS (Paris, France), using the cytoplasmic tail of mouse Shisa9/CKAMP44 (171‐424 AA), cloned into pB27 (N‐LexA‐bait‐C, Cter‐free) with an adult mouse brain cDNA library.

### Constructs

The CKAMP44 constructs used in this study are based on mouse SHISA9/CKAMP44 (Uniprot Q9CZN4; 424AA). All constructs were generated using standard cloning techniques. For full‐length FLAG‐ or MYC‐tagged CKAMP44 DNA constructs in the pCMV vector, we cloned the coding sequence for the FLAG tag (AAs DYKDDDDK) or the MYC tag (AAs EQKLISEEDL) behind the coding sequence for the first N‐terminal 29 AAs of CKAMP44 (including the signal peptide). The constructs containing the cytoplasmic tail of CKAMP44 express the following AA sequences as either EGFP or GST fusion proteins: pEGFP‐C1‐CKAMP44 (171‐424AA), pEGFP‐C1‐CKAMP44‐ΔC (171‐415AA), pEGFP‐C1‐CKAMP44‐ΔNΔC (199‐415AA), pGEX‐6PI‐CKAMP44 (186‐424AA), pGEX‐6PI‐CKAMP44‐ΔC (186‐415AA).

The constructs pCMV2A‐FLAG‐PSD‐95 and pCMV2A‐FLAG‐SAP102 were described previously 45, and the constructs pCMV2A‐FLAG‐PSD‐95‐PDZ3‐SH3‐GK and pCMV2A‐FLAG‐PSD‐95‐PDZ123mut have also been described 46; the constructs pCMV2A‐FLAG‐PSD‐95‐PDZ12mut and pCMV2A‐FLAG‐PSD‐95‐PDZ3mut were cloned in similar fashion using standard recombinant DNA technology. We cloned pCMV3A‐MYC‐PICK1 (Uniprot Q9NRD5, human, 415 AA) from human cDNA. A construct expressing FLAG‐MAGI1 (mouse, 1432 pcDNA3 flag MAGI1c) was obtained from Addgene (Cambridge, MA, USA; clone #10714). For the pEGFP‐C1‐PKCα‐WT construct in our study, we used pHACE‐PKCalphaWT (Addgene clone #21232, human, Uniprot P17252, 672 AA) as a cloning template, and for the kinase‐deficient PKCα mutant DN (K368R), we used pHACE‐PKCalphaDN (Addgene clone #21235). For generation of virus particles, FLAG‐CKAMP44 was cloned into a lentiviral shuttle vector under the control of a human synapsin‐1 promoter.

### Cells/treatment/transfection

COS‐7 cells (purchased from ATCC) were maintained in Dulbecco's modified Eagle's medium (DMEM; Lonza, Verviers, Belgium) supplemented with 10% FBS (Sigma‐Aldrich, Taufkirchen, Germany), 2 mm l‐glutamine and penicillin/streptomycin at 37 °C with 5% CO_2_. Transient transfections were performed using Lipofectamine 2000 (Thermo Fisher Scientific, Waltham, MA, USA) according to the manufacturer's recommendations. COS‐7 cells were treated with 1 μm PMA for 30 min. For the inhibitor experiment (Fig. 2D), cells were treated for 120 min with 3.5 μm or 7 μm PKC inhibitor GF109203X followed by 60‐min treatment with 0.2 μm PMA. Primary neuronal cultures were generated essentially as described previously 45, with minor modifications: for primary rat hippocampal neuronal cultures, embryonic E18 Wistar Unilever rats were used. Following decapitation, hippocampi from 5 to 10 pups were isolated and collected in ice‐cold DMEM (Lonza). Tissue was partially digested in trypsin/EDTA (Lonza) at 37 °C for 5 min. After stopping the reaction with 10% FBS (Biochrom, Berlin, Germany) in DMEM and subsequent washing in DMEM to remove traces of trypsin, the hippocampal tissue was suspended in neuron culture medium (Neurobasal A supplemented with B27 and 0.5 mm glutamine) and dissociated mechanically; neurons were then plated onto glass coverslips coated with a mixture of poly‐d‐lysine (Sigma) and laminin (Sigma) at a density of approximately 10^5^ cells per cm^2^. Cell debris was removed after healthy neurons adhered to the cover slips, and neurons were maintained for 2–4 weeks with minimal perturbation at 37 °C with 5% CO_2_ in neuron culture medium. Lentiviral particles were prepared by the NeuroCure Virus Core Facility essentially as described by Lois *et al*. 47. Primary neurons were transduced at DIV10–15 and analysed after fixation at DIV20–24. PMA treatment of neurons was with 0.2 μm PMA for 15 min. All animals used in this study were treated according to German regulations on the use of animals for research purposes and reported under the permit T0280/10.

### Coimmunoprecipitation

Shisa9/CKAMP44 proteins were overexpressed in COS‐7 cells together with their candidate interaction partners. Twenty‐four hours post‐transfection, cells were harvested in IP buffer [50 mm Tris pH 7.5, 100 mm NaCl, 0.1 – 1% Triton‐X and Mini Complete Inhibitors (Roche, Basel, Switzerland)] and lysed with 5–10 strokes using a 30‐G needle. Lysates were cleared by two centrifugations for 10 min at 20 000 ***g***. For IP, lysates were incubated for 3 h at 4 °C with 2 μg of the respective antibody and transfected proteins were pulled down with protein‐G agarose (Roche) for 1 h. Beads were washed three times for 5 min at 4 °C. Samples were analysed by SDS/PAGE and western blot (WB).

### GST pull‐down assays and GST protein purification

GST‐tagged proteins were expressed and purified according to the manufacturer's recommendations (GE Healthcare, Freiburg, Germany). Briefly, BL21 *Escherichia coli* transformed with the regarding GST protein constructs were cultured O/N. Expression of GST proteins was induced with 0.1 mm IPTG, and cells were lysed in TBS containing 1% Triton‐X. After lysate clearing at 20 000 ***g***, GST‐tagged proteins were purified using glutathione agarose (Thermo Fisher Scientific). For GST pull‐down assays, the GST‐tagged proteins (bound to the glutathione agarose beads) were incubated with lysate from adult mouse brains or from transfected COS‐7 cells. For brain lysate pull‐down experiments, adult C57Bl6 mice were sacrificed by cervical dislocation; whole brains were removed and placed directly in ice‐cold TN buffer [50 mm Tris, pH 7.5, 100 mm NaCl, and Mini Complete Inhibitors without EDTA (Roche)] and homogenised. Triton‐X was added to the lysate (final concentration of 1%) and lysates were kept on ice for 15 min. TN buffer was added to generate a final Triton‐X concentration of 0.5%. Cell debris was removed by high speed centrifugation (20 000 ***g***). After 3 h of incubation at 4 °C, the glutathione agarose beads were washed three times and the bound proteins were analysed by SDS/PAGE and WB. For the *in vitro* kinase assay, GST fusion proteins were purified using glutathione agarose and eluted with 50 mm glutathione in 200 mm Tris (pH 8). They were desalted using a Zeba Spin Desalting Column (40 K MWCO; Thermo Scientific).

### *In vitro* PKCα kinase assay

Purified and desalted GST‐CKAMP44 protein was used for an *in vitro* PKCα kinase assay (SignalChem, Richmond, Canada; #P61–18G) according to the manufacturer's instructions. Samples were analysed with Phos‐tag SDS/PAGE followed by WB.

### Phos‐tag SDS/PAGE

Shisa9/CKAMP44 phosphorylation was assessed using the Phos‐tag system (Wako, Neuss, Germany), which facilitates phosphorylation‐dependent retarded protein migration through a polyacrylamide gel, and subsequent WB. Phos‐tag gel electrophoresis (6–8% polyacrylamide supplemented with 50 μm Phos‐tag) was established using an optimised protocol with Zn^2+^ in a bis/tris‐buffered neutral pH gel system either as described previously 48 or according to the manufacturer's recommendations. Phos‐tag gels were generally blotted using a wet transfer blotting set‐up. As a control for phosphorylated proteins, the proteins were dephosphorylated using a thermosensitive alkaline phosphatase (FastAP; Thermo Fisher Scientific). The transfected cells were lysed in 1× FastAP buffer (Thermo Fisher Scientific) containing 1% Triton‐X and Mini Complete protease inhibitors without EDTA (Roche). The lysates were cleared by centrifugation and proteins were subsequently dephosphorylated for 2 h at 37 °C using FastAP.

### Immunofluorescence/colocalisation analysis

After 2–4 weeks in culture, differentiated neurons were fixed in 4% paraformaldehyde (PFA) in phosphate‐buffered saline (PBS) for 10 min and immunofluorescence was performed as described previously 49 according to standard IF protocols. Briefly, cells were permeabilised in 0.2% Triton‐X in PBS for 5 min, washed in PBS and blocked with 4% bovine serum albumin (BSA) in PBS for 1 h at room temperature. They were then incubated overnight with the primary antibodies in the same solution at 4 °C, washed with PBS and subsequently incubated with secondary antibodies in blocking solution. After final washes in PBS, coverslips were mounted with Fluoromount‐G (Southern Biotech, Birmingham, AL, USA). Images were acquired with a Leica (Wetzlar, Germany) laser‐scanning confocal microscope (Leica TCS‐SP5 II) using a 63× objective. Dendritic segments of rat hippocampal neurons were analysed for colocalisation of FLAG‐CKAMP44‐FL (cyan, Alexa488) with endogenous PICK1 (magenta, Alexa568). Regions of interest (ROIs) of merged images were selected blind to the conditions of the experiments and analysed with the imagej (NIH, Bethesda, MD, USA) plugin Puncta Analyzer as described previously 50. Number of cells (*n*) and number of cultures (*N*) are stated in the figure legend. FLAG‐CKAMP44‐FL puncta (cyan) were analysed for colocalised PICK1 (magenta). Data are normalised to the mean of the untreated condition and presented as mean ± SEM (standard error of the mean). Statistics were calculated using graphpad Prism 7 (GraphPad Software, La Jolla, CA, USA) (two‐tailed Mann–Whitney *U*‐test for unpaired data).

### Antibodies

Antibodies used in this study include GFP (mouse, Roche 11814460001; goat, Abcam, Cambridge, UK, AB6673, WB 1 : 5000), FLAG (mouse, rabbit, Sigma F1804 and F7425, respectively, WB 1 : 5000), FLAG‐HRP (Sigma A8592, WB 1 : 5000), MYC (mouse, Clontech, Mountain View, Canada, 631206; rabbit, Cell Signaling, Danvers, MA, USA, 2272S, WB 1 : 5000), MYC‐HRP (Abcam AB62928, WB 1 : 5000), GST (goat, GE Healthcare, 27457701V, WB 1 : 5000), CKAMP44 (rabbit, custom made by David's Biotechnology, Regensburg, Germany, WB 1 : 1000), actin (rabbit, Sigma A2066, WB 1 : 3000), GAPDH (rabbit, Abcam AB9485, WB 1 : 2000), GluA1 (rabbit, Millipore, Billerica, MA, USA, AB1504, WB 1 : 2000), MAP2 (rabbit, Millipore AB5622; guinea pig, Synaptic Systems, 188004), PICK1 (rabbit, Abcam AB3420, WB 1 : 2000), PKCα (mouse, Millipore 05–154, WB 1 : 2000), PSD‐95 (mouse, UC Davis/NIH NeuroMab Facility, Davis, CA, USA, 75–028, WB 1 : 2000), SAP102 (mouse, UC Davis/NIH NeuroMab Facility 75–058, WB 1 : 2000) and vGlut1 (mouse, UC Davis/NIH NeuroMab Facility 75–066). For coimmunoprecipitation experiments, 2 μg of the respective antibodies was used. Unspecific mouse IgGs (mIgG), as required (SantaCruz, Biotechnology, Dallas, TX, USA, SC‐2025, 2 μg), were used for negative controls in coimmunoprecipitation studies. All primary and secondary antibodies were diluted in 5% milk/PBST for WB or in 4% BSA/PBS for immunofluorescence experiments (1 : 1000). For immunofluorescence experiments, we used anti‐guinea‐pig‐Alexa405 (Abcam, ab175678), anti‐mouse‐Alexa488 and anti‐rabbit‐Alexa568 or anti‐mouse‐Alexa568 and anti‐rabbit‐Alexa488 (Thermo Fisher Scientific A‐21200 and A‐11036 or A‐11031 and A‐21441, respectively).

## Author contributions

SAK and SAS designed the study. SAK, NR and HZ performed experiments and collected the data. SAK, NR and SAS analysed data. SAK and SAS wrote the manuscript. All authors reviewed the manuscript.

## Supporting information


**Fig. S1.** PDZ‐dependent Shisa9/CKAMP44 protein–protein interactions.
**Fig. S2.** Shisa9/CKAMP44, PICK1 and PKC form a protein complex.
