# Supplementary material for: Protein kinase C regulates AMPA receptor auxiliary protein Shisa9/CKAMP44 through interactions with neuronal scaffold PICK1
Source: FEBS Open Bio. 2017 Aug 15;7(9):1234–45. doi: 10.1002/2211-5463.12261 (PMC5586339; doi:10.1002/2211-5463.12261)
Supplement: Supplementary file 1 — Fig. S1. PDZ‐dependent Shisa9/CKAMP44 protein–protein interactions. Fig. S2. Shisa9/CKAMP44, PICK1 and PKC form a protein complex. [file FEB4-7-1234-s001.pdf]

Supplementary Information

Figure S1 - PDZ-dependent Shisa9/CKAMP44 protein-protein interactions

A, B Coimmunoprecipitation experiments of PSD-95 (FLAG-PSD-95, A) or SAP102 (FLAG-SAP102, B) with the wild-type cytoplasmic tail of Shisa9/CKAMP44 (EGFP-CKAMP44) or with the cytoplasmic tail of Shisa9/CKAMP44 including a deletion of the PDZ ligand motif (EGFP-CKAMP44-ΔC, see also Figure 2A for a construct overview). Proteins were immunoprecipitated using a GFP antibody (GFP-IP) or mouse IgGs (mIgG-IP) as a negative control, and detected using the respective antibodies as indicated. Inputs (lysates) are shown on the left.

C, D GST-pull-down assays of different GST-tagged Shisa9/CKAMP44 proteins or GST alone (negative control) with adult mouse brain lysate. Precipitated proteins were detected by western blot. Endogenous PSD-95 (C) and SAP102 (D) interact with the cytoplasmic tail of Shisa9/CKAMP44 (GST-CKAMP44) but not with GST-CKAMP44-ΔC (with deletion of the PDZ-binding motif).

E Overview of PSD-95 constructs used for the interaction study with GST-tagged Shisa9/CKAMP44 variants.

F GST-pull-down assays (right panel) of GST-tagged CKAMP44, GST-CKAMP44-ΔC or GST alone (negative control) with COS-7 lysate after overexpressing different FLAG-PSD-95 constructs (see E). Precipitated proteins were detected by western blot with antibodies as indicated (PSD-95 top right panel; CKAMP44 pull-down control bottom right panel). Input controls are shown on the left; PSD-95 variants are detected with a FLAG antibody, CKAMP44 variants and GST control with a GST antibody.

Figure S2 – Shisa9/CKAMP44, PICK1 and PKC form a protein complex.

Coimmunoprecipitation experiments following expression of Shisa9/CKAMP44 (FLAG-CKAMP44-FL), PICK1 (MYC-PICK1), and PKCα (EGFP-PKCα-WT) in COS-7 cells indicate that the three proteins form a complex. After pull-down of either FLAG-CKAMP44-FL, MYC-PICK1 or EGFP-PKCα-WT with the respective antibodies, coprecipitated proteins are detected by western blot, as indicated. Mouse IgGs (mIgG) serve as a negative pulldown control.

Figure S1

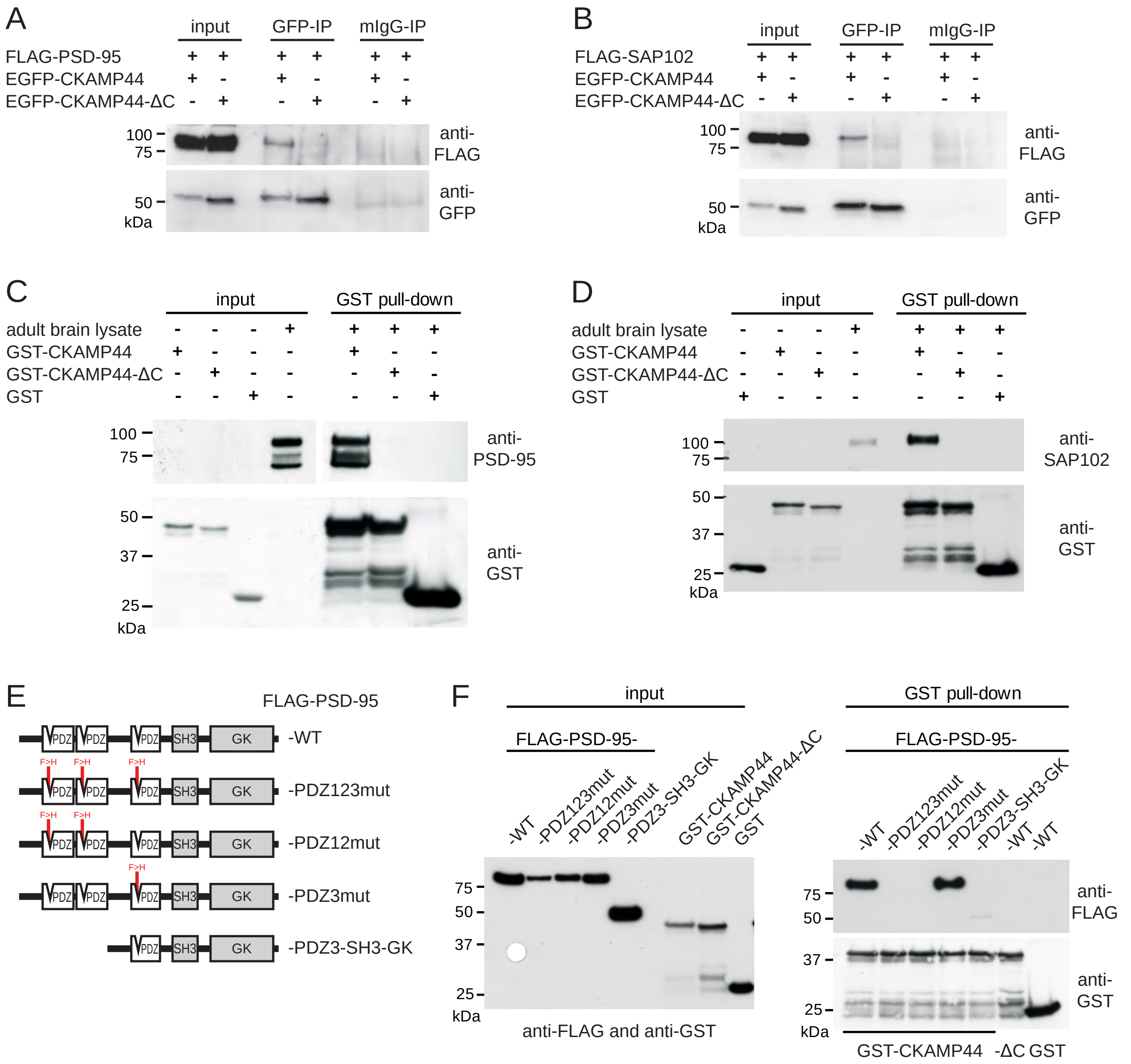

A
input
GFP-IP
mIgG-IP
FLAG-PSD-95
EGFP-CKAMP44
EGFP-CKAMP44-ΔC
100
75
50
kDa
anti-FLAG
anti-GFP
B
input
GFP-IP
mIgG-IP
FLAG-SAP102
EGFP-CKAMP44
EGFP-CKAMP44-ΔC
100
75
50
kDa
anti-FLAG
anti-GFP
C
input
GST pull-down
adult brain lysate
GST-CKAMP44
GST-CKAMP44-ΔC
GST
100
75
50
37
25
kDa
anti-PSD-95
anti-GST
D
input
GST pull-down
adult brain lysate
GST-CKAMP44
GST-CKAMP44-ΔC
GST
100
75
50
37
25
kDa
anti-SAP102
anti-GST
E
FLAG-PSD-95
PDZ
SH3
GK
F>H
-WT
-PDZ123mut
-PDZ12mut
-PDZ3mut
-PDZ3-SH3-GK
F
input
GST pull-down
FLAG-PSD-95-
-WT
-PDZ123mut
-PDZ12mut
-PDZ3mut
-PDZ3-SH3-GK
GST-CKAMP44
GST-CKAMP44-ΔC
GST
75
50
37
25
kDa
anti-FLAG and anti-GST
anti-FLAG
anti-GST
GST-CKAMP44
-ΔC
GST

Figure S2

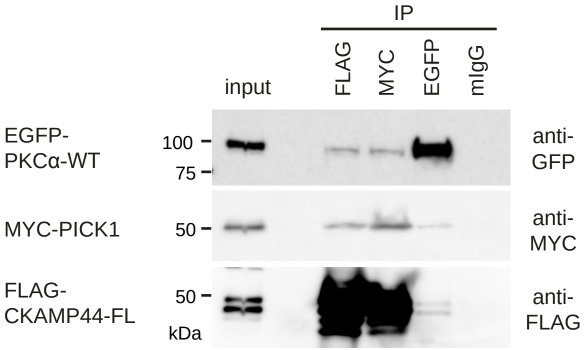

IP
FLAG
MYC
EGFP
mIgG
input
EGFP-
PKCα-WT
100
75
anti-
GFP
MYC-PICK1
50
anti-
MYC
FLAG-
CKAMP44-FL
50
kDa
anti-
FLAG
